# Prevalence, Risk Factors, and Human Health Implications of *Salmonella enterica* and *Campylobacter* spp. in Vermont Backyard Poultry

**DOI:** 10.1111/zph.70004

**Published:** 2025-07-29

**Authors:** Chelsey A. Patch, Katalin M. Larsen, Cheryl M. Armstrong, Siddhartha Kanrar, Alessandra M. Michaelides, Purna Chakraborty, Kelcey Harper, Valarie Devlin, Lorrie Martin, Alia Lunna, Hannah L. Blackwell, Sarah C. Nguyen, Anna Penny, Andrea J. Etter

**Affiliations:** ^1^ Department of Nutrition and Food Sciences The University of Vermont Burlington Vermont USA; ^2^ United States Department of Agriculture Agricultural Research Service Wyndmoor Pennsylvania USA; ^3^ Vermont Department of Health Laboratory Colchester Vermont USA

**Keywords:** antimicrobial resistance, backyard poultry, *campylobacter*, public health, *salmonella*, zoonoses

## Abstract

**Introduction:**

Backyard poultry (BYP) are increasingly linked to cases of campylobacteriosis and salmonellosis.

**Methods:**

Between 2022 and 2024, soiled bedding samples from 70 BYP farms were tested for *Campylobacter* spp. and/or *Salmonella enterica.*

**Results:**

Nine farms (12.86%) had at least one sample positive for *S. enterica,* while 19.05% (12/63) tested positive for *Campylobacter* spp. We sequenced 54 *S. enterica* isolates from eight farms in this sample and four farms from previous sampling in 2021 (*n* = 12 total farms) to determine the genetic characteristics of *S. enterica* from backyard poultry. *Salmonella* Schwarzengrund was the most common serovar (33%; 18/54) found, followed by Kentucky (16.7%; 9/54) and serovars Hadar (14.8%; 8/54) and Enteritidis (14.8%; 8/54). Though over half of isolates (51.9%; 28/54) exhibited no predicted genotypic or phenotypic resistance to antimicrobials, some serovars such as *Salmonella* Hadar were resistant to multiple antimicrobials. Four isolates had intermediate phenotypic resistance to ciprofloxacin and two were resistant to ampicillin.

**Conclusions:**

In summary, the frequency of *Campylobacter* and *Salmonella* in BYP populations of Vermont may pose a significant public health risk. Although the rate of antimicrobial resistance was low among *S. enterica* isolates, resistance to medically important antibiotics was observed, and isolate serovars aligned with serovars implicated in human illness in Vermont.

**Summary**

- Adult BYP in Vermont frequently carry *Salmonella* (13% of farms) and *Campylobacter* (19% of farms).
- *Salmonella* detected in BYP in Vermont matches *Salmonella* found in human illness outbreaks associated with BYP.
- *Salmonella* from BYP rarely has resistance to antibiotics used to treat human illness.

## 1 Introduction

*Campylobacter* spp. and non‐typhoidal *Salmonella* (NTS) cause an estimated 96 and 79 million global cases of foodborne illness annually, respectively (WHO 2015). *Campylobacter* spp. are Gram‐negative, microaerophilic curved rods with corkscrew motility and low environmental stress tolerance (Silva et al. 2011). Conversely, NTS are hardy and adaptable Gram‐negative rods with over 2500 known serovars belonging to two major species: *S. bongori* and *S. enterica* , the latter more commonly linked to human illness (WHO 2018). Most illnesses are self‐limiting, though complications include Guillain‐Barré syndrome from *Campylobacter* spp. and/or reactive arthritis and post‐infection inflammatory bowel syndrome from both bacteria (Scallan et al. 2015). Drug‐resistant *Campylobacter* spp. and *S. enterica* are classified as serious threats by the Centres for Disease Control and Prevention (CDC, n.d.), with specific *S. enterica* serovars such as Infantis being particularly concerning due to its resistance and increasing implications in human infections (CDC 2019; Majowicz et al. 2010; Tyson et al. 2021).

Although *S. enterica* is typically acquired from contaminated foods, live animal contact‐associated infections are increasing (Basler et al. 2016; Stapleton et al. 2024). Between 1990 and 2022, 141 outbreaks caused 10,496 infections linked to live poultry contact (Basler et al. 2016; Stapleton et al. 2024). The COVID‐19 pandemic heightened interest in home food production, spiking BYP ownership (privately owned, non‐commercial poultry) in the U.S., along with a concurrent spike in BYP‐associated illnesses in 2020 (Nichols et al. 2021; Niles et al. 2021; Stapleton et al. 2024). While data on the frequency of BYP ownership is scarce, a previous study in Vermont found that 23% of participants (*n* = 401/1730) currently owned or had owned BYP in the last year (Larsen et al. 2022).

In previous U.S. studies, *S. enterica* prevalence rates in BYP ranged from 2% to 19% (Clothier et al. 2018; Larsen et al. 2022; McDonagh et al. 2019). The lowest prevalence was 1.7%, found in California (44/2627 samples), followed by Massachusetts (2%; 1/53 flocks) and Washington (3%; 1/34 flocks). The highest prevalence was found in Vermont (19%; 8/42 flocks) and the Southeastern US (19%; 178/930 samples from 10 farms) (Clothier et al. 2018; Larsen et al. 2022; McDonagh et al. 2019; Parzygnat, Crespo, et al. 2024; Shah et al. 2020).

While live poultry‐associated campylobacteriosis outbreaks in humans are rarely reported, *Campylobacter* spp. are highly prevalent in poultry and pose a substantial burden to human health (Lin 2009; Taylor et al. 2013; Weis et al. 2016). Between 2016 and 2020, 17.3% of recorded *Campylobacter* spp. infections in Vermont (*n* = 166/962) were from patients with live poultry exposure (J. Brennan, personal communication, March 19, 2021), which increased to 20.5% in 2023 (M. Cahill, personal communication, April 18, 2024). Studies report *Campylobacter* spp. in 10%–86% of backyard flocks globally (Anderson et al. 2012; Keerthirathne et al. 2022). Detection among poultry is more likely among larger flocks, those near ruminants, with wet litter accumulation, poor cleaning and sanitation, mixed poultry species, and the presence of turkeys (Dermatas et al. 2024; El‐Tras et al. 2015; Mbai et al. 2022; Schweitzer et al. 2021).

Overall, data are limited on the prevalence of *S. enterica* and *Campylobacter* spp. in BYP. This study aimed to (i) assess the prevalence of *S. enterica* and *Campylobacter* spp. in Vermont BYP, (ii) identify risk factors for pathogen detection and (iii) assess the serovar distribution, antimicrobial resistance profiles, and understanding of human health impacts of *S. enterica* isolates.

## 2 Materials and Methods

Participants for this observational, cross‐sectional study were opportunistically recruited via Front Porch Forum (a Vermont online community forum), poultry swaps, and an agricultural fair. BYP owners in the state of Vermont who owned at least one bird of a poultry species were eligible to be included. During farm visits, ≥ 50 g of soiled bedding (wood shavings, sawdust, hay, straw, etc.) and faeces were aseptically collected from multiple areas in the poultry enclosure (e.g., under roosts, within nesting boxes and near indoor feeders), along with basic farm information (Figure S1). At poultry swaps, bedding and/or faeces in carrying cages were collected along with farm information from the seller.

### 2.1 Microbiological Sample Analysis and Molecular Confirmation

#### 2.1.1 *Campylobacter* spp.

Samples were immediately processed or preserved in Cary Blair transport medium (Becton, Dickinson and Company, Franklin Lakes, NJ). A 1:4 ratio of soiled bedding to Bolton Broth with laked horse blood was aseptically transferred into Whirl‐pak bags (Nasco, Fort Atkinson, WI), hand‐massaged for two minutes, transferred into screw top tubes, and incubated (42°C; 28–48 h). Samples were tested for *Campylobacter* spp. using the BAX System Real‐Time PCR Assay for *Campylobacter jejuni* /*coli*/*lari* via BAX Q7 thermocycler (Hygiena, Camarillo, CA). Additionally, 100 μL Bolton broth was streaked onto chromogenic *Campylobacter* plating medium (R&F Products, Downers Grove, IL) or *Campylobacter* blood agar plates (BAP), incubated (42°C; 48 h) in microaerophilic conditions using AnaeroPack jars and gas‐generating satchels (Mitsubishi Gas Chemical Company Inc., Tokyo, Japan), and cryogenically preserved at −80°C in 10% skim milk. Due to rare isolation from young birds (USDA‐NIFA n.d.), *Campylobacter* spp. were not tested for among birds under 4 weeks old.

#### 2.1.2 *Salmonella enterica*

Samples were immediately processed or stored at −20°C until processing. Twenty‐five grams of sample and 100 mL of buffered peptone water (BPW) (BD Difco, Franklin Lakes, NJ or Thermo Scientific, Waltham, MA) were transferred aseptically into Whirl‐pak bags, hand‐massaged for two minutes, and incubated (37°C; 4 h). Then, 1 mL was transferred to 10 mL of Tetrathionate (TT) broth (BD Difco, Franklin Lakes, NJ) and incubated (37°C; 4 h; 200 rpm). TT inoculum (20–40 μL) was streaked onto *Salmonella* Chromogenic Plating medium (SCPM) (R&F Products, Downers Grove, Illinois) or xylose‐lysine‐tergitol 4 (XLT‐4) plating medium (BD Difco, Franklin Lakes, NJ or Thermo Scientific, Waltham, MA) and incubated (35°C, 24 h for SCPM; 37°C, 24–48 h for XLT‐4). Up to four colonies from each presumptive positive sample were isolated and cryopreserved at −80°C in 25% glycerol (Thermo Scientific, Waltham, MA). Molecular confirmation was performed via *hilA* PCR as previously described (Larsen et al. 2022; Pathmanathan et al. 2003).

### 2.2 Whole Genome Sequencing (WGS) and Genomic Analysis

Fifty‐four PCR confirmed *S. enterica* isolates (representing 21 samples and 12 farms) were sequenced, with samples coded for anonymity.

Twenty‐four isolates were transported on ice to the Vermont Department of Health Laboratory (VDHL, Colchester, VT) and bioinformatic analysis and quality determination were performed as described in Table 1. Thirty‐three isolates were shipped as frozen stocks to the USDA Agricultural Research Station (USDA‐ARS) in Wyndmoor, Pennsylvania. One colony per isolate was grown in Brain Heart Infusion broth at 35°C (170 rpm) for 18 h. Subsequently, 8.5 mL was pelleted at 4500 rpm in a 50 mL conical tube, and the pellet was resuspended in 1 mL phosphate buffer saline (PBS). The solution was transferred to a 2 mL tube, centrifuged, and the pellet was resuspended in 0.5 mL of DNA/RNA shield (Zymo Research, Irvine, CA). Samples were shipped to Plasmidsaurus Inc. where the DNA was sequenced using PCR‐free Oxford Nanopore Technology's V14 chemistry with MinION R10.4.1 flow cell (Oxford, United Kingdom). Three isolates were sequenced by both the USDA and VDH. Genome assembly was completed as described in Table 1.

**TABLE 1:** Whole genome sequencing (WGS) and genomic analysis methods for *S. enterica* isolates.

	VT department of health (Colchester, VT)	USDA‐ARS (Wyndmoor, PA)
No. of Isolates	24	33
Sub‐culture conditions	Tryptic Soy Agar with 5% Sheep Blood agar (Northeast Laboratory Services, Winslow, ME) at 35°C ± 2°C for 18–24 h	Brain Heart Infusion broth at 35°C with agitation for 18 h
Isolate confirmation	MALDI‐TOF MS (Bruker Daltonics, Billerica, MA) via an Extended Direct Transfer method with identifications performed using MBT Compass software and reference library (Bruker Daltonics). Isolates with initial identification scores < 2.00 underwent MALDI‐TOF MS using an Extraction method and/or by API 20E (bioMerieux, Marcy l'Etoile, France).	N/A
DNA extraction	18–24 h cultures extracted via the QIAamp DNA Mini Kit (QIAGEN, Germantown, MD)	Performed by Plasmidsaurus Inc. (Eugene, OR) with cultures shipped in DNA/RNA shield as per their protocol
Quantification	Qubit 3 fluorometer (Invitrogen, Carlsbad, CA)	Performed by Plasmidsaurus Inc.
Library prepararion	Illumina DNA Prep Kit (Illumina Inc., San Diego, CA)	PCR‐free Oxford Nanopore Technology's V14 chemistry
Sequencing	Illumina MiSeq (v2, 300 cycles) and Illumina iSeq 100 (Illumina Inc)	MinION R10.4.1 flow cell (Oxford, United Kingdom)
Bioinformatic analysis/quality determination	PulseNet 2.0 Platform ((CDC); Atlanta, GA)	Plasmidsaurus Inc.
*De novo* assembly	SPAdes v3.15.5 (Andrey Prjibelski et al. 2020)	Flye v.2.9.1 (Kolmogorov et al. 2019) or Canu v2.2 (Koren et al. 2017)
Genome circularization	Not done	Circlator v.1.5.5 (Hunt et al. 2015)
Species identification	ANI using mummer v3.23	ANI using mummer v3.23
Contamination check	MIDAS v1.3.2 (Kurtz et al. 2004; Nayfach et al. 2016)	Not done
Allele calls and plasmid finder	BLAST v2.12 (Altschul et al. 1990)	PlasmidFinder v2.1 (Carattoli et al. 2014)
Pathogenicity Islands	SPIFinder2.0 software (Roer et al. 2016)	SPIFinder2.0 software (Roer et al. 2016)
AST profile	NCBI‐amrfinderplus v3.9.3 (Feldgarden et al. 2021)	ResFinder v4.3.1 (Bortolaia et al. 2020)
Serotyping	SeqSero2 v1.2.1 (Zhang et al. 2019)	SeqSero2 v1.1.0 (Zhang et al. 2019)
Average *de novo* coverage	≥ 30×	~150×
Average quality‐Q score	≥ 30	≥ 20
Assembly length	4.4–5.7 Mb	4.8–5.2 Mb
Secondary species abundance	≤ 1.0	NA
Core percent called	≥ 85	NA
NCBI accession	PRJNA675595	PRJNA1112792

### 2.3 Antimicrobial Minimum Inhibitory Concentration (MIC)

MICs for all 54 isolates were determined using Sensititre plates (NARMS Gram‐Negative CMV4AGNF AST Plate; TREK Diagnostic Systems Incorporated, Cleveland, OH) per manufacturer instructions, with classifications (sensitive/intermediate/resistant) based on standard CLSI breakpoints (CLSI 2022) or standard National Antimicrobial Resistance Monitoring System for Enteric Bacteria (NARMS) standards (CDC 2025).

### 2.4 Ethics Statement

We did not directly use animal subjects for this study, but one isolate from farm 29 was derived from cloacal swabs as described in (Larsen et al. 2022). That work was performed under IUCAC approval PROTO202000013 by the University of Vermont. We did not have control groups for this earlier study, as we could not be sure ahead of time which chickens would or would not carry Salmonella. Our laboratory safety procedures were approved by the University of Vermont IBC committee under REG201900012.

### 2.5 Statistical Analysis

Nine categorical criteria were tested for association with bacterial carriage (e.g., detection of *S. enterica* or *Campylobacter* spp.) at the farm level (i.e., ‘owner’): owner's farm setting (rural/semi‐rural/urban), season (winter/spring/summer/fall), collection type (farm visit/poultry swap/agricultural fair [CVF]), chickens only presence (yes/no), multiple poultry species (yes/no), farm size (small: 1–9 birds/medium: 10–25/large: 26–300), housing type (inside an outbuilding only ‘indoor’/free‐range/penned access to outdoors), mixed age (yes/no), bird age category (chicks: < 16 weeks/adults: ≥ 16 weeks/mixed). The questionnaire used for data collection is shown in Figure S1. Urban/rural classification was based on proximity to undeveloped/forested areas and other homes/businesses. ‘Chickens’ indicated layers or dual‐purpose breeds, while ‘broilers’ indicated meat birds. ‘Chicks’ were < 16 weeks, the standard pre‐egg production age (Siceloff et al. 2022). Three numeric criteria tested included average age of birds, flock size, and species count.

Analyses were conducted in R Studio (v 2021.09.2). Little's test showed data were not missing completely at random (MCAR; *p* = 0.00038), thus random forest‐based imputation was used. Fisher's exact tests for categorical variable pre‐selection, followed by multivariable logistic regression to determine risk factors at the farm‐level, were conducted. Significant associations of pre‐selected variables (*p* < 0.05 for *S. enterica* , *p* < 0.01 for *Campylobacter* spp.) were modelled in logistic regressions, with goodness of fit assessed by Hosmer and Lemeshow χ^2^ tests. This approach adjusted for potential confounding bias, though residual confounders cannot be completely ruled out. Log odds were transformed to odds ratios with 95% confidence intervals (CI), and log odds with *p* < 0.05 were considered risk factors.

## 3 Results and Discussion

### 3.1 Sample Demographics

**FIGURE 1:**
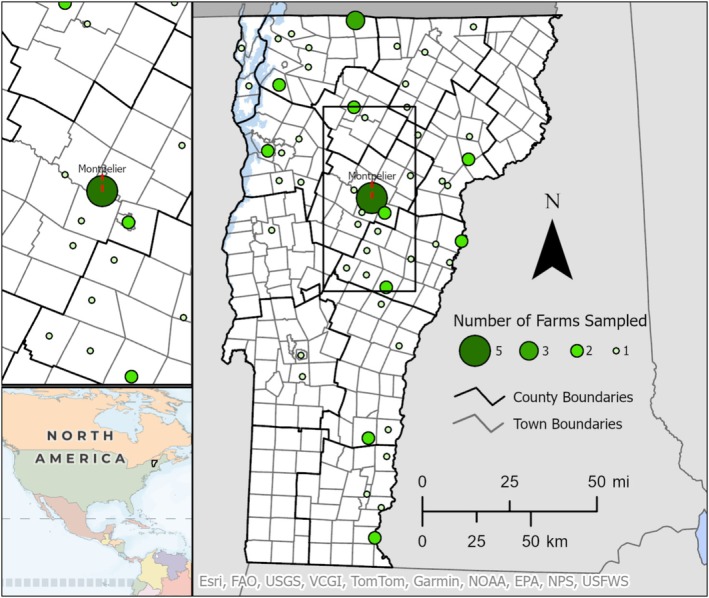
Sampling map showing geographical location of backyard flocks sampled in 2024. Location was indicated by the coordinates of the town centre nearest the farm sampled. Map courtesy of Harrison Shukei.

Between February 2022 and May 2024, 70 BYP farms (where ‘farm’ is defined as one or more samples of poultry belonging to an individual BYP owner, regardless of whether actual sampling took place on farm, at the agricultural fair, or at a poultry swap) were sampled (Figure 1). Not all demographic information was available for all farms. Most farms (78.46%, *n* = 51/65) raised chickens (layers and/or broilers) exclusively. On farms with other poultry, ducks were most common (15.38%, *n* = 10/65), followed by geese (7.69%, *n* = 5/65), turkeys (6.15%, *n* = 4/65), and quail (4.62%, *n* = 3/65). Flocks were typically kept in penned enclosures (50.0%, *n* = 30/60) or free‐range (35.0%, *n* = 21/60), with fewer indoors‐only (10.0%, *n* = 6/60) or mixed housing (5.0%, *n* = 3/60). Most BYP were sampled via farm visits (57.97%, *n* = 40/69); others were sampled at poultry swaps (31.88%, *n* = 22/69), agricultural fair (7.25%, *n* = 5/69), or a combination (2.90%, *n* = 2/69). Sampling occurred predominantly in the fall (63.77%, *n* = 44/69), followed by summer (20.29%, *n* = 14/69), spring (10.14%, *n* = 7/69), and winter (4.35%, *n* = 3/69); one farm was tested in spring and summer (1.45%). Most farms were rural (77.78%, *n* = 49/63), followed by semi‐rural (20.63%, *n* = 13/63), and urban (1.59%, *n* = 1/63). Typically, adult birds were kept (64.0% of farms), *n* = 32/50, but 24.0% had chicks (*n* = 12/50), and 12.0% had both (*n* = 5/60). The average bird age was 523 days (just under 18 months). The median flock size was 12, with a mode of 7.

### 3.2 Prevalence of *S. enterica* and *Campylobacter*

**FIGURE 2:**
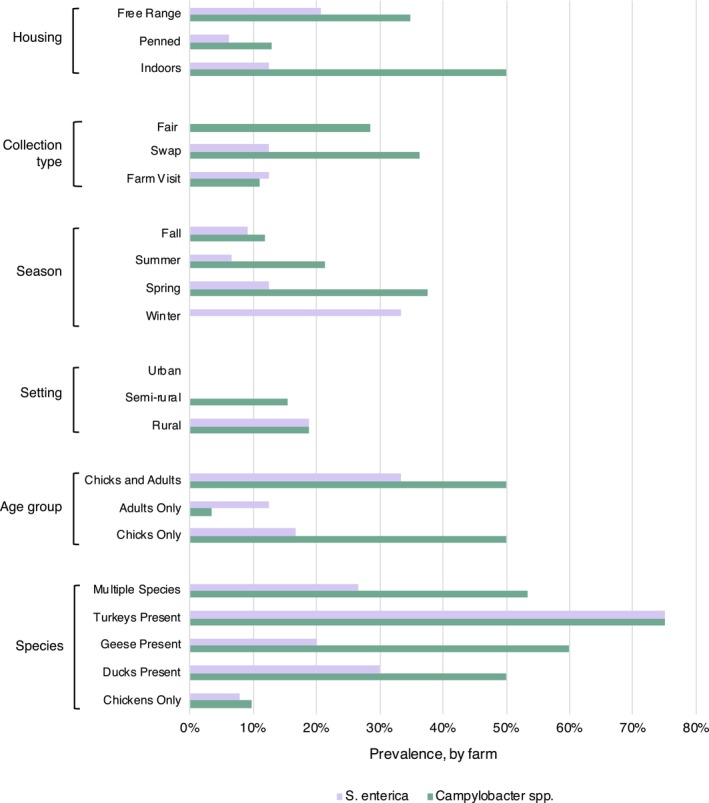
Characteristics of farms positive for *S. enterica* and/or *Campylobacter* spp. Characteristics included bird housing type, collection type (fair = local agricultural fair, swap = poultry swap), season, farm setting, age, and birds' species. Data shows the prevalence of either bacterium, given the characteristic (e.g., 19.05% (4/21)) farms with chickens only harboured *S. enterica*. The data represents the 9/70 farms positive for *S. enterica* and 12/63 farms positive for *Campylobacter* spp. Note that not all farms had all data available.

A total of 203 samples were taken from 63 farms and tested for *Campylobacter* spp.; farm‐level prevalence via qPCR was 19.05% (12/63 farms), as farms with birds under 4 weeks (7/70) were not tested for *Campylobacter* spp. (USDA‐NIFA n.d.). A total of 265 samples from 70 farms were tested for *S. enterica* , which was detected via isolation followed by PCR on 12.86% (9/70) of farms. Figure 2 shows general farm characteristics for positive samples. Both bacteria were detected on three farms: two large, diversified farms and one with mixed age ducklings.

*S. enterica* prevalence was lower than earlier data (2019–2021) from Vermont (Larsen et al. 2022) and the Southeastern US (19%) (Parzygnat, Crespo, et al. 2024), but higher than studies in Massachusetts (3%) and Ontario, Canada (3%) (Brochu et al. 2019; McDonagh et al. 2019). Vermont flocks had a lower average age (17 months; 40.7% under 12 months) compared to Massachusetts flocks (25 months; 33.4% under 12 months), and lower poultry age is a known risk factor for infection (Keerthirathne et al. 2022; Koutsoumanis et al. 2019; McDonagh et al. 2019). Variation could stem from our prevalence calculation by farm, while other studies used sample (Parzygnat, Crespo, et al. 2024), bird (Brochu et al. 2019), or flock (Larsen et al. 2022; McDonagh et al. 2019). Sample type also varies: other studies used post‐mortem cecal samples (Brochu et al. 2019), litter grab samples and cloacal swabs (Larsen et al. 2022), or a mix of faecal, bedding, soil samples, and environmental swabs (Parzygnat, Crespo, et al. 2024). Additionally, husbandry and housing characteristics differ widely, complicating BYP studies.

The lower prevalence of *S. enterica* (12.86%) compared to *Campylobacter* spp. (19.05%) aligns with previous studies (Brochu et al. 2019; Parzygnat, Crespo, et al. 2024; Parzygnat, Dunn, et al. 2024) and Vermont human salmonellosis (average of 20/year) and campylobacteriosis (average of 33/year) cases with reported live poultry contact (M. Cahill, personal communication, April 18, 2024; J. Brennan, personal communication, March 19, 2021). However, *Campylobacter* spp. prevalence found (19.05%) is on the lower end of the reported global rate of 10%–86% in BYP (Anderson et al. 2012; Brochu et al. 2019; Keerthirathne et al. 2022; Parzygnat, Dunn, et al. 2024; Pohjola et al. 2016; Santos‐Ferreira et al. 2022). Prevalence was lower (19% vs. 35%) than a study in Ontario, Canada; however, Brochu's study had a substantial number of turkey samples, which are known to have higher *Campylobacter* spp. rates (Brochu et al. 2019; Schweitzer et al. 2021). It was, however, similar to the Southeastern US (21%); (Heuer et al. 2001; Parzygnat, Dunn, et al. 2024; Rivoal et al. 1999). The fastidious nature of *Campylobacter* spp. and inherent challenges with recovering viable organisms from the environment may have constituted a limitation of this study (Silva et al. 2011).

### 3.3 Risk Factors Associated With *Campylobacter* spp. Carriage at the Farm Level

Potential variables following Fisher's Exact tests associated with *Campylobacter* spp. carriage (Table S1) included season (*p* = 0.009); collection type (farm visit/poultry swap/fair; *p* = 0.011); chickens only (*p* = 0.001) versus multiple poultry species present (*p* = 0.0002); farm size (*p* = 0.003); housing type (*p* = 0.017); and bird age (*p* = 0.0005). Variables with a *p* < 0.001 were modelled in a logistic regression (*n* = 5; Table S2). Multi‐age flocks were a risk factor compared to only adults (OR = 26.98, 95% CI [1.43, 510.46], *p* = 0.028), as were young birds (OR = 21.5, 95% CI [1.67, 276.45] *p* = 0.019). For every day older a flock was, the odds of *Campylobacter* spp. detection decreased by 0.4% (OR = 0.996, 95% CI [0.993, 0.997]; *p* = 0.03), which contradicts typical associations with older birds (Lin 2009). Farms with non‐chicken species had higher odds (OR = 10.97, 95% CI [1.16, 71.43], *p* = 0.036), and each additional poultry species increased the odds by a factor of 4.82 (95% CI [1.34, 17.31], *p* = 0.016).

Overall, risk factors for *Campylobacter* spp. carriage included multi‐age flocks, younger flocks, and multi‐species flocks, the latter being a known risk factor for *Campylobacter* detection among BYP (Schweitzer et al. 2021). Multi‐age flocks, in particular may facilitate inter‐flock transmission, with older birds passing *Campylobacter* on to younger, previously uninfected birds or new, *Campylobacter‐*positive birds introducing the pathogen to a previously uninfected flock. To reduce bacterial introduction, BYP owners could adopt the USDA‐FSIS recommendations for commercial poultry and use an ‘all in all out’ strategy (i.e., cull all birds on farm at once, and then wait a period of time and disinfect before purchasing new birds) (Keerthirathne et al. 2022), although this would be inconvenient and may not be feasible for BYP owners.

### 3.4 Risk Factors Associated With *S. enterica* Carriage at the Farm Level

Only ‘season’ showed a potential association with *S. enterica* carriage (*p* = 0.046) and was included in the multivariate logistic regression model. The model demonstrated that the odds of detecting *S. enterica* during winter were 34.56 times greater (OR: 34.56; 95% CI [1.86, 640.04], *p* = 0.017) than in fall, though only three farms were sampled in the winter, so this should be interpreted with caution. For every additional species present, the odds of detecting *S. enterica* increased 7.63‐fold (95% CI [1.05, 55.69], *p* = 0.045). For every day older a flock was, the odds of detecting *S. enterica* decreased by 0.3% (OR = 0.997, 95% CI [0.994, 0.999], *p* = 0.046). Although *S. enterica* is typically isolated from poultry in warmer months and BYP‐associated illnesses peak in spring and summer (CDC 2022; Meher et al. 2022; Sivaramalingam et al. 2013; Stapleton et al. 2024; Van Der Fels‐Klerx et al. 2008), one study in Japan found higher rates from broiler meat in winter (Ishihara et al. 2020). Geography, management, and *S. enterica* fitness contribute to variability. The presence of multiple species was a risk factor for *S. enterica* ; birds of different species often come from different sources, increasing transmission risk (Behravesh et al. 2014). Additionally, younger flocks are more likely to carry *S. enterica* due to heightened susceptibility to colonisation and frequent exposure at the hatchery (Basler et al. 2014, 2016; McDonagh et al. 2019; Shaji et al. 2023).

### 3.5 Distribution of *S. enterica* Serovars

**FIGURE 3:**
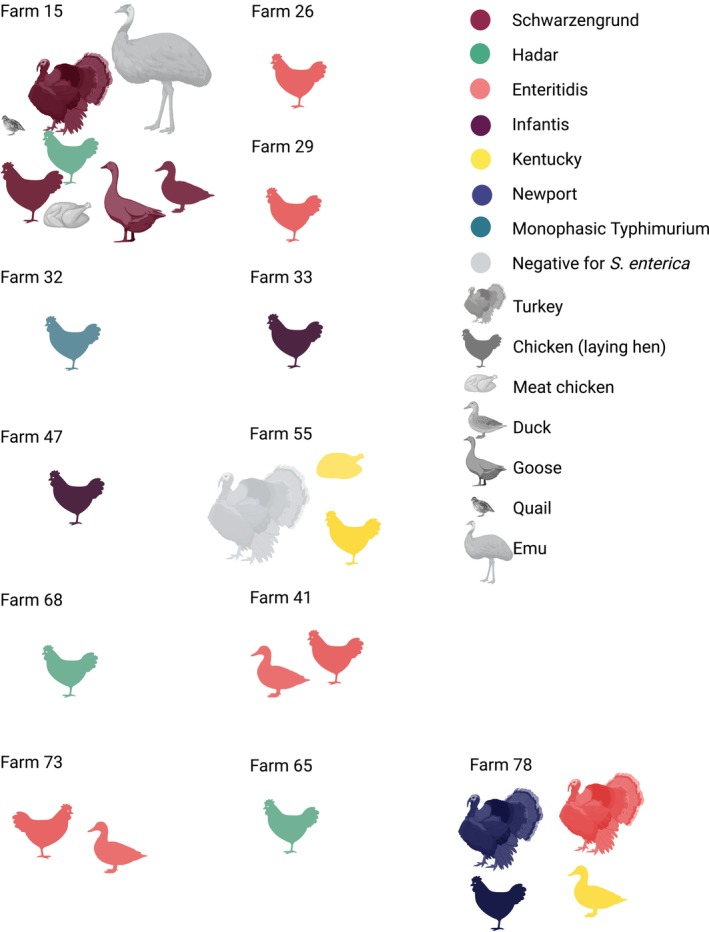
*Salmonella* serovars species distribution in sequenced samples from Table 2. *S. enterica* was often detected on farms with multiple poultry species. Isolates were enriched from soiled bedding samples from pens of the different poultry species, with the exception of Farm 29, which was sampled in 2021 using cloacal swabs. Not all species in a farm were positive for *S. enterica*, and in some farms, multiple serovars were present across or within species.

We sequenced 54 *S. enterica* isolates from 21 samples across 12 farms, including four farms from 2021 (Table 2; Table S3), and identified seven serovars. *Salmonella* Schwarzengrund was most common (33.3%, 18/54), followed by Kentucky (16.7%, 9/54), Enteritidis and Hadar (both 14.8%, *n* = 8/54), and Newport (13.0%, *n* = 7/54). Serovars Infantis and I 4:i:‐ (monophasic Typhimurium) were each found twice. Ten farms carried one serovar, and two farms carried multiple serovars, both with at least two poultry species (Figure 3). *Salmonella* Infantis, monophasic Typhimurium, and Hadar were found in layers. Enteritidis, Kentucky and Newport were found among multiple species, and Schwarzengrund was found among a variety of species. In two mixed‐species groups with Schwarzengrund, pens had been previously flooded by a severe weather event.

**TABLE 2:** Overview of farms with *S. enterica* isolates sequenced for this study.

Farm	Years	Samples taken	Positive samples	*S. enterica* isolates sequenced	Serovars detected	*Campylobacter* present?	Study sampling was associated with
15	2022–2024	135^a^	7	21	Schwarzengrund Hadar	Yes	This study (2022–2024) (Larsen et al. 2022)
26	2021	1	1	1	Enteritidis	Not tested	(Larsen et al. 2022)
29	2021	15^b^	1	1	Enteritidis	Not tested	(Larsen et al. 2022)
32	2021	1	1	2	I 4,[5],12:i:—	Not tested	(Larsen et al. 2022)
33	2021	1	1	1	Infantis	Not tested	(Larsen et al. 2022)
41	2022	1	1	1	Enteritidis	Not tested	This study
42B	2022	1	1	0^c^	NA	Not tested	This study
47	2022	1	1	1	Infantis	No	This study
55	2022	3	2	5	Kentucky	Yes	This study
65	2022	1	1	1	Hadar	No	This study
68	2022	1	1	1	Hadar	No	This study
73	2023	1	1	4	Enteritidis	Yes	This study
78	2023	6	3	12	Newport Enteritidis Kentucky	No	This study
*n* = 13		*n* = 168	*n* = 21	*n* = 54			

*Note:* Fifty‐four isolates were sequenced for this study, including five isolates from farms sampled in Larsen et al. 2022. One farm (42B) sampled for this study did not have an isolate available for sequencing due to technical issues with sample preservation. During sampling, for each positive sample, up to four isolates were preserved. These were PCR‐confirmed. Of the PCR‐confirmed isolates for each sample, at least one was sequenced.

^a^ Sampling was also performed in 2021 but there were no positive samples that year (Larsen et al. 2022).

^b^ These samples were cloacal swabs (Larsen et al. 2022).

^c^ Sample was lost due to technical issues.

Detection of Enteritidis and Infantis is noteworthy, as these serotypes are commonly associated with BYP‐linked human illness and have been targeted by FSIS for regulation in commercial poultry flocks (Stapleton et al. 2024; USDA‐FSIS 2022). Infantis is often outcompeted by better‐growing serovars in traditional culture‐based methods, meaning its prevalence may be underreported (Obe et al. 2023). From 2016 to 2020, Vermont reported 26 human cases (5.1% of cases) of *Salmonella* Infantis infection, with five (4.7%) also reporting contact with live poultry (J. Brennan, personal communication, March 19, 2021). *Salmonella enteritidis* is a top contributor to human illness (Stapleton et al. 2024; Tack et al. 2019), and a concern in egg production (Gantois et al. 2009). In Vermont, it accounted for 25.5% of NTS infections from 2016 to 2020 (J. Brennan, personal communication, March 19, 2021), with 48.1% (*n* = 51/130) of infected individuals reporting live poultry contact (J. Brennan, personal communication, March 19, 2021). *Salmonella* Hadar caused the largest U.S. BYP outbreak (Stapleton et al. 2024) and is a serovar of concern in turkey products (Anonymous 2024). In Vermont, only three cases of *Salmonella* Hadar were reported from 2016 to 2020, but two also reported live poultry contact (J. Brennan, personal communication, March 19, 2021). Conversely, 21 cases of *Salmonella* Newport were reported in Vermont from 2016 to 2020, with only six reporting live poultry contact (J. Brennan, personal communication, March 19, 2021). These findings suggest that serovars found in human illnesses with reported live poultry contact were commonly isolated from BYP flocks, supporting a live poultry infection route.

### 3.6 Salmonella Pathogenicity Island (SPI) Identification

**FIGURE 4:**
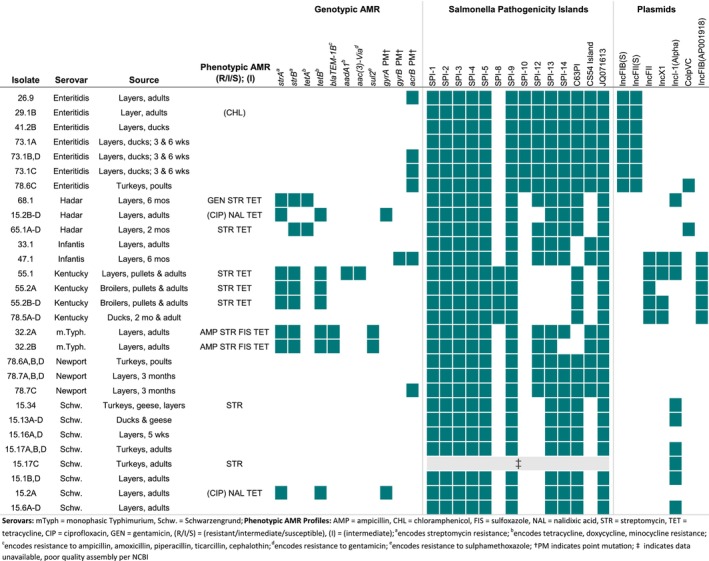
Phenotypic and genotypic AMR profiles, and presence of *Salmonella* Pathogenicity Islands (SPIs) and plasmids among sequenced *S. enterica* isolates. Key details (serovar, bird species, and age) were included. Combining isolates from the same sample with identical phenotypic and genotypic characteristics yielded 29 unique isolate profiles from 54 sequenced isolates. Resistance predictions for genotypic AMR were derived from ResFinder 4.0 results, although *aac(6′)‐Iaa* presence was not reported, as it is known to be cryptic and non‐functional (Bortolaia et al. 2020; Magnet et al. 1999). Not all predicted phenotypes were tested by the Sensititre phenotypic test plate used.

All isolates tested contained SPI‐1 and SPI‐2 (Figure 4), which are both associated with the type III secretion system (T3SS) and highly ubiquitous (Figueira and Holden 2012; Lou et al. 2019). All isolates also contained SPI‐3, SPI‐4, and SPI‐5, which play a lesser role in virulence (Rychlik et al. 2009), and SPI‐9, which is associated with the T1SS (Parkhill et al. 2001). Isolates carried either SPI‐8 (all Kentucky) or SPI‐13 in the same genomic location as in typhoidal serovars, though they have different functions (Espinoza et al. 2017). SPI‐10 was conserved in Enteritidis isolates, as previously documented (Saroj et al. 2008), while SPI‐12, associated with bacterial survival (Tomljenovic‐Berube et al. 2013), was found among all isolates except Kentucky and Schwarzengrund. SPI‐14, which may regulate SPI‐1 (Hu et al. 2024), was found in all but Kentucky and one monophasic Typhimurium isolate. No isolates contained SPI‐7 (specific to serovars Typhi, Dublin, and Paratyphi; Pickard et al. 2003), SPI‐11 (associated with *Salmonella choleraesuis* ), or SPI‐6 and SPIs 15–17 (associated with *Salmonella Typhi* ; Chiu et al. 2005; Vernikos and Parkhill 2006). Additionally, 92.3% of isolates (all except monophasic Typhimurium and Infantis) contained the C63PI iron transport system on SPI‐1 (Zhou et al. 1999). All contained an unnamed hit (accession number: JQ071613) on SPI‐2, which contains the *ssaD* gene (Bhowmick et al. 2011). Enteritidis, Newport, monophasic Typhimurium, and Infantis isolates all contained the CS54 island, contributing to *S. enterica* colonisation (Kingsley et al. 2003).

### 3.7 Plasmid Identification

At least one plasmid or incompatibility group was identified in most isolates (Figure 4). Type F plasmids (IncFIB(S) and IncFII(S)) were present in all eight Enteritidis isolates, while IncFIB and IncFII were found among all Kentucky isolates and one Infantis isolate (47.1). These plasmids are often linked to resistance to multiple antibiotics including tetracyclines, aminoglycosides, beta‐lactamases, and fluoroquinolones (McMillan et al. 2020), though our isolates only encoded tetracycline and streptomycin resistances. IncI(Alpha) was the second most common incompatibility group, found among Hadar, Kentucky, Infantis, and Schwarzengrund. IncI1 plasmids are associated with transferring to a broad range of hosts and antimicrobial resistance (AMR) genes in poultry and were found in BYP‐associated Hadar outbreaks from 2014 to 2020 (McMillan et al. 2020; Webb et al. 2022). All Kentucky isolates except one (55.2A) contained IncX1, associated with beta‐lactamase and aminoglycoside resistance, but it is naturally repressed (McMillan et al. 2020). ColpVC was present in all four Hadar isolates from farm 65 and one Enteritidis isolate (78.6C), though it can be cryptic (Oladeinde et al. 2018).

### 3.8 Antimicrobial Resistance (AMR) *S. enterica* Isolates

The most common resistance genes were for streptomycin (*strA, strB*) and tetracycline (*tetA, tetB*) (Figure 4). Cryptic gene *aac(6′)‐Iaa* was detected in 37/54 isolates but does not confer resistance and was excluded from further analysis (Feldgarden et al. 2021; Magnet et al. 1999). AMR‐conferring point mutations (Table S4) included T255S (*n* = 2) in *parC*; F28L (*n* = 7), L40P (*n* = 7), and A94T (*n* = 1) in *acrB*; S83Y (*n* = 4) and a frameshift at 734 leading to a premature stop codon at 818 (*n* = 1) in *gyrA*; and Q624K (*n* = 1) in *gyrB*. All missense mutations in *gyrA* and *gyrB* are associated with quinolone resistance (Campos Granados et al. 2023; Yang et al. 2023), and F28L and L40P in *acrB* are associated with macrolide resistance (MIC not tested) (Nuncio et al. 2022). At least one AMR gene or point mutation conferring resistance was found in all Kentucky, Hadar, Infantis, monophasic Typhimurium, and Newport isolates tested. Point mutations conferring resistance were most common among Kentucky, Newport, Hadar, Enteritidis, and Infantis isolates.

MIC testing revealed 18/54 isolates had resistance to at least one antimicrobial. Both monophasic Typhimurium isolates had five AMR genes (*strA, strB, tetB, blaTEM‐1B*, and *sul2*) encoding resistance to ampicillin, streptomycin, tetracycline, and sulfoxazole. One Kentucky isolate (55.1) contained five plasmid‐encoded genes (*aac(3)‐*VIa, *aadA1, strA, strB, and tetB*) and showed phenotypic resistance to streptomycin and tetracycline. Phenotypic and genotypic resistance matched in most isolates. Three Hadar isolates contained *strA*, *tetB*, and *gyrA* point mutation S38Y, exhibiting resistance to nalidixic acid and tetracycline, as well as intermediate ciprofloxacin resistance, but no streptomycin resistance. Two Schwarzengrund isolates (15.34 and 15.17C) exhibited phenotypic resistance (MIC = 32 mg/L) to streptomycin but carried no AMR genes. However, streptomycin is the most common antibiotic with discordant phenotypic: genotypic results (Neuert et al. 2018), and breakpoints for *S. enterica* streptomycin resistance have historically varied from 16 to 64 mg/L (Doran et al. 2006). Genes *tetA, tetB*, and *sul2* and resistance to tetracycline and streptomycin are common in *S. enterica* —a result of regular use of these antibiotics in food production (Pavelquesi et al. 2021; Pitti et al. 2023). Both ampicillin (considered essential) and ciprofloxacin are commonly used to treat salmonellosis (CDC 2019), making ampicillin resistance and intermediate ciprofloxacin resistance in BYP concerning. Overall, *S. enterica* from Vermont BYP have varying levels of AMR, from none to multidrug resistance.

This study had several limitations. First, we were limited to owners willing to have their poultry tested, which is an inherent challenge to studying backyard poultry. In the risk factor modelling, potential confounding and small sample size could have impacted odds ratio values. However, according to our previous study's statewide survey (Larsen et al. 2022), 23% of Vermont households have backyard poultry. Based on the census results of 2022 which indicate 277,090 households (Anonymous 2022), that is 63,731 backyard flocks. A sample size calculation using a 19% prevalence for *S. enterica* and a 95% confidence interval with 10% precision yielded a sample size of 59.21. Our sample size of 70 flocks is larger than any other farm‐based BYP studies, which sampled 10–51 flocks (McDonagh et al. 2019; Parzygnat, Dunn, et al. 2024; Shah et al. 2020). The only larger sample sizes came from Veterinary Diagnostic centres which received dead birds for necropsy (Brochu et al. 2019; Clothier et al. 2018) Given the difficult‐to‐reach population and resources needed for sampling on property, testing, and whole genome sequencing, we believe these parameters and our sample size were appropriate. Third, we used two sequencing methods; however, Illumina and high‐coverage Nanopore MinION sequencing have equivalent accuracy for serotyping (Wu et al. 2021; Xu et al. 2020), AMR gene detection (Ye et al. 2024), and plasmid detection/sequencing (Lemon et al. 2017). We also confirmed AMR genotypes with phenotypic testing. Finally, we had significant challenges with culturing *Campylobacter* spp. from soiled bedding samples, despite repeated consultations with *Campylobacter* experts. This may have been due to *Campylobacter's* known sensitivity to oxidative stress, and it may be a challenge inherent to the sample type. Regardless, we were unable to grow *Campylobacter* spp. in the lab, and therefore unable to sequence it or perform AMR testing. Nonetheless, this work provides valuable insights into *Campylobacter* frequency and *S. enterica* frequency, serovars, and AMR among BYP.

## 4 Conclusions

This study found 12.86% *S. enterica* prevalence and 19.05% *Campylobacter* spp. prevalence on Vermont BYP farms. Risk factors for flock infection with either pathogen included owning multiple species, while having a mixed age flock was a risk factor for *Campylobacter* spp. infection. The *S. enterica* serovars detected are linked to human salmonellosis, posing a public health risk. While AMR was generally low, two isolates were resistant to ampicillin and four exhibited intermediate ciprofloxacin resistance. Overall, our findings demonstrate that *Campylobacter* spp. and *S. enterica* are prevalent among Vermont BYP, consist of serovars commonly associated with illness, adverse health outcomes, and outbreaks. Education on enhanced biosecurity for poultry owners is crucial to reduce bacterial contamination at the farm level and reduce illness among humans.

## Conflicts of Interest

The authors declare no conflicts of interest.

## Supporting information


**Figure S1.** Farm sampling sheet used to collect data. Participants' responses to the open‐ended questions were documented by the researchers, providing the opportunity for detailed, narrative descriptions.

**Table S1.** Farm characteristics tested via Fisher's Exact Test for association with *S. enterica infection, Campylobacter* spp. *infection*, and either *S. enterica* or *Campylobacter* spp. infection. The first category listed for each variable is the reference category.

**Table S2.** Odds ratios of specific variables with *p*‐values of less than 0.05 were tested in multivariate logistic regression for association with *S. enterica* carriage, *Campylobacter* spp. carriage, and detection of either bacterium.

**Table S3.** NCBI genome accession numbers for *S. enterica* isolates from this study.

**Table S4.** Point mutations conferring resistance were found in 62.9% (*n* = 34/54) of BYP *S. enterica* isolates, most commonly conferring quinolone resistance.

## Data Availability

The data that support the findings of this study are openly available in NCBI Bioproject at https://www.ncbi.nlm.nih.gov/bioproject, reference number PRJNA1112792 and PRJNA675595.
