# Supplementary material for: Prevalence, Risk Factors, and Human Health Implications of Salmonella enterica and Campylobacter spp. in Vermont Backyard Poultry
Source: Zoonoses Public Health. 2025 Jul 29;72(7):654–68. doi: 10.1111/zph.70004 (PMC12508789; doi:10.1111/zph.70004)
Supplement: Supplementary file 1 — Figure S1. Farm sampling sheet used to collect data. Participants' responses to the open‐ended questions were documented by the researchers, providing the opportunity for detailed, narrative descriptions. [file ZPH-72-654-s002.docx]

Backyard Poultry Sampling Information

Etter Lab Research Study 2023


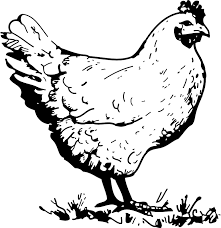


Date__________________

| Owner Name (First Last) |  |
| --- | --- |
| Owner Contact Information |  |
| Location of farm (nearest town) |  |
| Rural/urban/semi-rural |  |
| Number of birds in flock: |  |
| Age(s) of chickens (e.g., adult, pullet, chick) |  |
| Housing type (indoor only; indoor w/ penned run; free range, or other housing type) |  |
|  |  |
| Breeds: |  |
|  |  |
|  |  |
|  |  |
|  |  |

**Labeling Sample Campylobacter Testing?**

☐ “Adult” (16 wks and over) **☐** YES (4 weeks or older)

☐ “Chick” (under 16 weeks) ☐ NO (under 4 weeks)
