# Supplementary material for: Prevalence, Risk Factors, and Human Health Implications of Salmonella enterica and Campylobacter spp. in Vermont Backyard Poultry
Source: Zoonoses Public Health. 2025 Jul 29;72(7):654–68. doi: 10.1111/zph.70004 (PMC12508789; doi:10.1111/zph.70004)
Supplement: Supplementary file 2 — Table S1. Farm characteristics tested via Fisher's Exact Test for association with S. enterica infection, Campylobacter spp. infection, and either S. enterica or Campylobacter spp. infection. The first category listed for each variable is the reference category. [file ZPH-72-654-s005.docx]

Table S1. Farm Characteristics Tested for Association with S. enterica infection, Campylobacter spp. infection, and either S. enterica or Campylobacter spp. infection. The first category listed for each variable is the reference category.

| Variable Tested for Association with Bacterial Infection | P-Value for Fisher’s Exact | | |
| --- | --- | --- | --- |
|  | ***S. enterica*** | ***Campylo-bacter* spp.** | **Either Bacteria** |
| Setting  (rural/semi-rural/urban) | 0.287 | 1 | 0.486 |
| Season  (winter/spring/summer/fall) | 0.046* | 0.009** | 0.042* |
| Collection type  (farm visit/poultry swap/Champlain Valley Fair) | 1 | 0.011* | 0.040* |
| Only chickens present on farm  (yes/no) | 0.071 | 0.001** | 0.005** |
| Multiple species present on farm  (yes/no) | 0.091 | 0.0002** | 0.002** |
| Farm size category  (small: 1-9/medium: 10-25/large: 26-300) | 0.619 | 0.003** | 0.005** |
| Housing type  (indoor/free range/penned) | 0.145 | 0.017* | 0.039* |
| Mixed age  (yes/no) | 1 | 0.212 | 0.786 |
| Age category of birds  (chicks: under 16 weeks/adults:16 weeks and above/chicks and adults) | 0.166 | 0.0005** | 0.005** |
| Coinfection  (yes/no) | 0.1774 | | n/a |

*Note:* Data demonstrate that overall, there are numerous characteristics possibly associated with infection of either bacterium, though there were a higher number of significant variables associated with *Campylobacter* spp. infection. The only variable that was found to be significant for its potential association with *S. enterica* and *Campylobacter* spp. infection was season.

*P-value of less than 0.05; **P-value of less than 0.01
