# Supplementary material for: Prevalence, Risk Factors, and Human Health Implications of Salmonella enterica and Campylobacter spp. in Vermont Backyard Poultry
Source: Zoonoses Public Health. 2025 Jul 29;72(7):654–68. doi: 10.1111/zph.70004 (PMC12508789; doi:10.1111/zph.70004)
Supplement: Supplementary file 3 — Table S2. Odds ratios of specific variables with p‐values of less than 0.05 were tested in multivariate logistic regression for association with S. enterica carriage, Campylobacter spp. carriage, and detection of either bacterium. [file ZPH-72-654-s001.docx]

Table S2. Odds ratios of specific variables with p-values of less than 0.05 tested in multivariate logistic regression for association with S. enterica carriage, Campylobacter spp. carriage, and detection of either bacterium.

| Risk Factor | | *S. enterica* | | *Campylobacter* spp. | | Either Bacteria | |
| --- | --- | --- | --- | --- | --- | --- | --- |
|  |  | **p-Value** | **Odds Ratio**  [95% CI] | **p-Value** | **Odds Ratio** [95% CI] | **p-Value** | **Odds Ratio** [95% CI] |
| *Categoric* | **Season:** Winter *(ref. category: fall)* | 0.017* | 34.56  (1.86-640.04) | *ns* | *ns* | *ns* | *ns* |
|  | **Chickens Only:** No *(ref. category: yes)* | *ns* | *ns* | 0.036* | 10.97 (1.16-71.43) | *ns* | *ns* |
|  | **Age Category:** Chick Only *(ref. category: adult only)* | *ns* | *ns* | 0.019* | 21.5 (1.67-276.45) | 0.019* | 11.67  (1.50-90.82) |
|  | **Age Category:** Chick and Adult | *ns* | *ns* | 0.028* | 26.98 (1.43-510.46) | 0.009** | 43.33  (2.59-724.69) |
| *Numeric* | **Number of Species** | 0.045* | 7.63  (1.05-55.69) | 0.016* | 4.82 (1.34-17.31) | 0.016* | 4.74  (1.33-16.89) |
|  | **Average Days Old** | 0.046* | 0.997 (0.994-0.999) | 0.030* | 0.996 (0.993-0.999) | 0.013* | 0.997(0.995-0.999) |

*P-value of less than 0.05; **P-value of less than 0.01; *ns* = non-significant
