# Supplementary material for: Prevalence, Risk Factors, and Human Health Implications of Salmonella enterica and Campylobacter spp. in Vermont Backyard Poultry
Source: Zoonoses Public Health. 2025 Jul 29;72(7):654–68. doi: 10.1111/zph.70004 (PMC12508789; doi:10.1111/zph.70004)
Supplement: Supplementary file 5 — Table S4. Point mutations conferring resistance were found in 62.9% (n = 34/54) of BYP S. enterica isolates, most commonly conferring quinolone resistance. [file ZPH-72-654-s004.docx]

**Table S2.** **Point mutations conferring resistance in *S. enterica* isolates from backyard poultry in Vermont.** At least one point mutation conferring resistance was found in 62.9% (n = 34/54) of isolates. Point mutations most commonly found have potential associated resistance to quinolones, followed by macrolides.

| Gene | Mutation | Frequency  (n isolates, serovar) | Potential Associated Resistance |
| --- | --- | --- | --- |
| *acrB* | F28L, L40P  A94T | Enteritidis (5/x)  Infantis (1/x)  Newport (1/x)  Enteritidis (1/x) | Macrolides  (Nuncio et al., 2022) |
| *gyrA* | S83Y  Frameshift at 734 🡪 premature stop codon at 818 | Hadar (3)  Schwarzengrund (1/x)  Enteritidis (1/x) | Quinolones  (Yang et al., 2023) |
| *gyrB* | Q624K | Infantis (1/x) | Quinolones  (Campos Granados, 2023) |
| *parC* | T255S  T57S | Infantis (1/x)  Newport (1/x)  Kentucky (9/x)  Newport (7/x)  Hadar (5/x)  Infantis (2/x) | Quinolones  (Campos Granados, 2023)  Quinolones  (Campos Granados, 2023) |

**References**

Campos Granados, C. M., Sierra Gómez Pedroso, L. del C., Hernández-Pérez, C. F., Ballesteros-Nova, N. E., Rubio-Lozano, M. S., Sánchez-Zamorano, L. M., & Delgado-Suárez, E. J. (2023). Strong antibiotic resistance profiles in Salmonella spp. isolated from ground beef in Central Mexico. *Veterinaria México OA, 10*. Retrieved from https://doi.org/10.22201/fmvz.24486760e.2023.1215

Nuncio, A. S. P., Webber, B., Pottker, E. S., Cardoso, B., Esposito, F., Fontana, H., . . . Rodrigues, L. B. (2022). Genomic characterization of multidrug-resistant Salmonella Heidelberg E2 strain isolated from chicken carcass in southern Brazil. *Int J Food Microbiol, 379*, 109863. doi:10.1016/j.ijfoodmicro.2022.109863

Yang, X., Yang, S., Liu, S., Liu, S., Zhang, J., Guo, W., . . . Wu, Q. (2023). Characterization of quinolone resistance in Salmonella enterica serovar Typhimurium and its monophasic variants from food and patients in China. *Journal of Global Antimicrobial Resistance, 35*, 216-222. doi:https://doi.org/10.1016/j.jgar.2023.09.010
